# Rhizospheric Bacterial Strain *Brevibacterium casei* MH8a Colonizes Plant Tissues and Enhances Cd, Zn, Cu Phytoextraction by White Mustard

**DOI:** 10.3389/fpls.2016.00101

**Published:** 2016-02-16

**Authors:** Tomasz Płociniczak, Aki Sinkkonen, Martin Romantschuk, Sławomir Sułowicz, Zofia Piotrowska-Seget

**Affiliations:** ^1^Department of Microbiology, University of Silesia in KatowiceKatowice, Poland; ^2^Department of Environmental Sciences, University of HelsinkiLahti, Finland; ^3^Institute of Environmental Sciences, Kazan Federal UniversityKazan, Russia

**Keywords:** phytoextraction, PGPE, heavy metals, *Brevibacterium*, *Sinapis alba* L.

## Abstract

Environmental pollution by heavy metals has become a serious problem in the world. Phytoextraction, which is one of the plant-based technologies, has attracted the most attention for the bioremediation of soils polluted with these contaminants. The aim of this study was to determine whether the multiple-tolerant bacterium, *Brevibacterium casei* MH8a isolated from the heavy metal-contaminated rhizosphere soil of *Sinapis alba* L., is able to promote plant growth and enhance Cd, Zn, and Cu uptake by white mustard under laboratory conditions. Additionally, the ability of the rifampicin-resistant spontaneous mutant of MH8a to colonize plant tissues and its mechanisms of plant growth promotion were also examined. In order to assess the ecological consequences of bioaugmentation on autochthonous bacteria, the phospholipid fatty acid (PLFA) analysis was used. The MH8a strain exhibited the ability to produce ammonia, 1-amino-cyclopropane-1-carboxylic acid deaminase, indole 3-acetic acid and HCN but was not able to solubilize inorganic phosphate and produce siderophores. Introduction of MH8a into soil significantly increased *S. alba* biomass and the accumulation of Cd (208%), Zn (86%), and Cu (39%) in plant shoots in comparison with those grown in non-inoculated soil. Introduced into the soil, MH8a was able to enter the plant and was found in the roots and leaves of inoculated plants thus indicating its endophytic features. PLFA analysis revealed that the MH8a that was introduced into soil had a temporary influence on the structure of the autochthonous bacterial communities. The plant growth-promoting features of the MH8a strain and its ability to enhance the metal uptake by white mustard and its long-term survival in soil as well as its temporary impact on autochthonous microorganisms make the strain a suitable candidate for the promotion of plant growth and the efficiency of phytoextraction.

## Introduction

The continued industrialization of countries has led to extensive environmental problems that induce the contamination of soil and water (Rajkumar and Freitas, 2008). Among the pollutants, heavy metals pose a critical concern to human health and the environment (Rajkumar et al., 2009a). Due to the widespread contamination, searching for innovative ways to remove metals from the environment has become a priority in the remediation field (Rajkumar et al., 2009b; Ali et al., 2013). One of the most promising strategies is phytoextraction, which is defined as the use of plants to take up pollutants from contaminated soil (Salt et al., 1998; Glick, 2010; Płociniczak et al., 2013b). The success of metal extraction depends on many factors, but the most important are a plant’s ability to uptake and translocate metals to its stems and leaves, metal bioavailability and soil type (Glick, 2010; De-Bashan et al., 2012; Płociniczak et al., 2013a). The results of many studies have shown that the efficiency of heavy metal phytoextraction may be supported by metal-resistant bacteria that belong to the plant growth-promoting bacteria group (PGPB; Glick, 2005; Jing et al., 2007; Li and Ramakrishna, 2011; Li et al., 2012; Sun et al., 2014). PGPB include both rhizospheric (PGPR) and endophytic (PGPE) bacteria (Ma et al., 2011a). Because of the extensive root exudation of many easily degradable compounds, the rhizosphere is a nutrient-rich environment for beneficial bacteria that may colonize the internal tissues of plants and exist as endophytes. Although bacterial plant growth-promoting endophytes (PGPEs) exist in plants to varying degrees, and despite the fact that they are transient, they are often capable of triggering physiological changes that promote the growth and development of plants, even those that are growing in metal-contaminated soil (Rajkumar et al., 2009a).

Generally, the beneficial effects of endophytes are greater than those of many rhizobacteria and these might be enhanced when a plant is growing under either biotic or abiotic stress conditions. It has been shown that PGPE may confer plants with a higher tolerance to heavy metal stress and may stimulate the growth of the host plant through several mechanisms (Ma et al., 2011b). That is one reason why PGPE apart from their application as biocontrol agents and biofertilizers are used to enhance *in situ* phytoextraction (Chen et al., 2010; Becerra-Castro et al., 2011). Heavy-metal-resistant endophytes can enhance plant growth, decrease metal phytotoxicity and affect metal translocation and accumulation in plants and thus play a significant role in the adaptation of plants to a polluted environment (Sessitsch et al., 2013). The inoculation of plants with PGPR/PGPE has been shown to enhance the growth of plants and their development in heavy metals and/or soils contaminated with organic pollutants (Becerra-Castro et al., 2011; Thavasi et al., 2014). Generally, PGPB interact directly or indirectly with a host plant through several mechanisms. These mechanisms involve the production of indole acetic acid (IAA), phytohormones, 1-aminocyclopropane-1-carboxylic acid deaminase (ACCD) and biosurfactants (Arshad et al., 2007; Gravel et al., 2007; Juwarkar et al., 2008; Shoebitz et al., 2009; Li et al., 2012; Sun et al., 2014; Thavasi et al., 2014). Moreover, metal-resistant microorganisms have been shown to increase the availability of heavy metals in soil through soil acidification by producing siderophores and/or by mobilizing metal phosphates (Abou-Shanab et al., 2008; Jing et al., 2012; Płociniczak et al., 2013b).

The aim of this study was to estimate the potential of a metal-resistant strain of *Brevibacterium casei* MH8a to enhance Zn, Cd, and Cu uptake by white mustard under laboratory conditions. Moreover, its potential to promote the growth of plants was also determined. Additionally, the ability of MH8a to colonize the internal tissues of *Sinapis alba* and the ecological consequences of its introduction into soil on autochthonous bacterial communities were also determined.

## Materials and Methods

### Isolation and Identification of Metal-Resistant Bacteria

The *B. casei* MH8a strain was chosen from 12 strains isolated from metal-contaminated rhizosphere soil of *S. alba* L. collected around a non-ferrous steelworks in Dkabrowa Górnicza, Upper Silesia, Poland, which contained the following concentrations of heavy metals: Zn 926, Cd 32, Cu 60, and Ni 150 mg kg^–1^ dry weight (dw). The metal-resistant bacteria were isolated on a 10% Tryptic Soy Agar (TSA) medium that was supplemented with 5 mM of zinc as ZnCl_2_. The plates were incubated at 28°C for 7 days. Isolated strains were identified using the MIDI microbial identification system (MIDI, Newark, DE, USA) according to the producer’s procedure. Identification of MH8a strain was also confirmed based on 16S rRNA gene sequence analysis. For 16S rRNA gene amplification, the universal bacterial primers 8F (5′ AGTTTGATCATCGCTC AG 3′) and 1492R (5′ GGTTACCTTGTTACGACTT 3′) targeting fragment size 1484 bp were used (Pacwa-Płociniczak et al., 2014). The obtained sequence was compared to known 16S rRNA gene sequences using the BLAST server at the National Center for Biotechnology Information (NCBI; https://blast.ncbi.nlm.nih.gov/Blast.cgi?PAGE_TYPE~=~BlastSearch).

DNA sequences were aligned using ClustalW. Phylogenetic analyses were performed by the neighbor–joining (NJ) method to test the support for the phylogeny with a bootstrap analysis based on 1,000 replicates using MEGA software ver. 7.0.

### Determination of Metal Minimal Inhibitory Concentration (MIC)

To assess the level of the resistance to heavy metals of the tested strain, the minimal inhibitory concentration (MIC) of Zn, Cu, and Cd was estimated. The MIC values were determined in triplicate in an MES buffered minimal medium (MBMM) as described by Rathnayake et al. (2013). The MBMM medium was supplemented with an increasing content of the metals (1–10 mM of Zn and Cu and 0.5–3 mM of Cd). The MIC was defined as the lowest metal concentration at which bacterial growth was not observed.

### Evaluation of Plant Growth-Promoting Activities

Secretion of siderophores by the tested strain was detected using the method described by Schwyn and Neilands (1987) using blue agar plates that contained Chrome azurol S dye (CAS). Orange zones around the colonies on blue agar were considered to be a positive reaction that indicated the production of siderophores.

1-Aminocyclopropane-1-carboxylic acid deaminase activity was assayed according to a modified Honma and Shimomura (1978) method as described by Saleh and Glick (2001). ACCD activity was expressed in nmol of α-ketobutyrate mg^–1^ h^–1^. The protein concentration of microbial cell suspensions was determined using the Bradford (1976) method.

Indole acetic acid production was assessed using Salkowski’s reagent according to the modified method of Bric et al. (1991). The IAA concentration in cultures was calculated using the calibration curve of pure IAA (1–100 μg mL^–1^ of medium) as the standard.

The phosphate solubilizing ability of the tested bacteria was determined on a National Botanical Research Institute’s phosphate (NBRIP) agar medium as described by Płociniczak et al. (2013a).

The production of hydrogen cyanide by MH8a was tested using the Lorck (1948) method as shown in Pawlik and Piotrowska-Seget (2015).

The bacterial isolate was tested for the production of ammonia in peptone water according to Cappuccino and Sherman (1992).

### Selection of a Rifampicin-Resistant Mutant of MH8a

A spontaneous mutant of the MH8a was selected by plating on a Luria Bertani (LB) medium that was amended with 5 μg mL^–1^ of rifampicin. Next, the growing colony was reinoculated on LB plates with a successively higher content of rifampicin (up to 150 μg mL^–1^ of medium). The stability of rifampicin resistance was confirmed by subculturing MH8a^rf^ five times on an LB medium without antibiotic selection. The cultures were stored in 20% glycerol at -80°C (Płociniczak et al., 2013b). MH8a^rf^ had the same biochemical features as the parental MH8a and was used in pots experiment.

### Inoculum Preparation

For soil inoculation, the metal-resistant MH8a^rf^ strain was cultured on a Luria-Bertani (LB) medium on an orbital shaker at 120 rpm (28°C) for 24 h. The number of bacteria in the inoculum was established based on the turbidimetry and plating methods. The appropriate volume of bacterial culture was centrifuged (6000 rpm, 21°C, 20 min); the harvested bacteria were washed twice with sterile distilled water and resuspended in 50 mL sterile water.

### Experimental Set-Up

The phytoextraction experiment was conducted in laboratory conditions using sandy loam contaminated with heavy metals collected in the vicinity of the non-ferrous steelworks “Mikrohuta” in Dkabrowa Górnicza. Selected physicochemical properties of the tested soil used in the experiments were as follows: pH (H_2_O) 5.4 ± 0.1; organic matter 9.6 ± 0.2 g kg^–1^; total N 284 ± 14 mg kg^–1^; total P 170 ± 6 mg kg^–1^; Fe 8792 ± 42 mg kg^–1^; Cd 32 ± 2.1 mg kg^–1^; Cu 60 ± 4.6 mg kg^–1^; Zn 926 ± 32 mg kg^–1^; Ni 150 ± 11 mg kg^–1^.

The experiment had a completely randomized block design with three replications that had two treatments (I) plants growing in soil inoculated with the tested MH8a^rf^ strain and (II) control – plants growing in soil inoculated with distilled water that contained thermal inactivated MH8a^rf^ cells instead of the suspension of living bacteria.

Prior to the experiment, the soil was kept for 2 days at room temperature and sieved. Then the soil moisture was adjusted and maintained at 20% which corresponded to 50% of the maximum water holding capacity of the soil. Seeds of *S. alba* L. cv. Nakielska were placed in pots (total volume of 500 mL) that contained 400 g of the soil and kept in a growth room under controlled light (14-h photoperiod at 15,000 lux; temperature 23/18°C light/dark). After 2 weeks, 50 mL of the bacterial solution was poured into the soil up to the number of 10^8^ cells of the tested strains g^–1^ dw soil. Plants (five plants per pot) were grown in the conditions described above for 28 days.

### Effect of the Bacterial Inoculant on Plant Biomass and Metal Accumulation

After 28 days of incubation, the plants were removed from the pots and shoots (stems and leaves) and roots were weighed separately. Before weighing, the roots were washed carefully using distilled water. The fresh and dw of shoots and roots as well as heavy metal accumulation in the above and underground parts were measured. The metal concentration in the shoots and roots of *S. alba* was determined in triplicate using atomic absorption spectrometry as described in Płociniczak et al. (2013b). The usefulness of *S. alba* in phytoextraction was confirmed by estimating the translocation factor (TF) for the tested metals (Yoon et al., 2006).

### Effect of the Bacterial Inoculant on the Structure of Bacterial Community

Changes in the biodiversity and community structure of the autochthonous bacterial populations after soil inoculation with the MH8a^rf^ strain were determined in triplicate at 24 h, 7 and 28 days using the phospholipid fatty acid (PLFA).

Phospholipid fatty acids were isolated from 2 g of fresh soil as described by Pennanen et al. (1999). The fatty acid methyl esters were separated using a gas chromatograph (Hewlett-Packard 6890, USA) with an HP-Ultra 2 capillary column (25 m, 0.22 mm ID) and hydrogen as the carrier gas. PLFA compounds were detected using a flame ionization detector (FID) and identified using MIDI Microbial Identification System software (Sherlock TSBA 6 method and TSBA 6 library; MIDI, Inc., Newark, DE, USA).

### Survival of MH8a^rf^ in Soil and its Ability to Colonize Plant Tissues

The number of living MH8a^rf^ cells in soil was determined at 24 h, 7 and 28 days after soil inoculation. In order to estimate the colony-forming units (cfu) of bacteria in soil and plant tissues, the dilution-plate method on Luria-Bertani (LB) agar with the addition of 150 μg mL^–1^ of rifampicin was used. As a control, a suspension of soil inoculated with thermal inactivated MH8a^rf^ cells was plated on LB agar with the addition of an antibiotic.

Plant colonization by MH8a^rf^ was determined 24 h, 7 and 28 days after soil inoculation with the tested strain. Roots, stems and leaves were surface sterilized with 70% ethanol (2 min), 5% sodium hypochlorite (2 min), and 10% hydrogen peroxide (2 min). The samples were rinsed three times in sterile distilled water to remove the disinfectant. The sterilization process was verified by a plating final wash onto a TSA medium. Plates were incubated at 28°C for 7 days. If no microbial growth was found, the surface-sterilization process was recognized as being successful. The roots, stems, and leaves were macerated separately in 5 mL of 0.9% NaCl using a mortar and pestle and a 100 μL suspension was plated onto a TSA medium that contained rifampicin at a concentration of 150 μg mL^–1^ of medium and incubated at 28°C for 7 days (Kukla et al., 2014). At the same time, rifampicin-resistant bacteria were isolated from the control plants grown in soil inoculated with thermal inactivated MH8a^rf^ strain.

### Statistical Analysis

Statistical analysis was performed using STATISTICA 10.0 PL software (StatSoft, Tulsa, OK, USA). Analysis of variance (ANOVA) followed by a *post hoc* Least Significant Difference test were conducted in order to identify any significant effects of the introduced strains on the plant biomass as well as on the accumulation of heavy metals in the shoots and roots of *S. alba*. Differences to from the control plants with *p* < 0.01 in the plant inoculation experiments were considered to be significant. For the pot experiments, data were represented as the mean ± standard deviation (SD) of three replicates. For the PLFA experiments principal component analysis (PCA) was used. One-way multivariate analysis of variance (MANOVA) of the PCA-axes values and *post hoc* LSD tests (*p* < 0.05) were applied for the statistical testing of the separation of the profiles along each PC. For the PLFA experiment, data were represented as the mean ± standard deviation (SD) of three replicates.

## Results

### Identification of Isolate and MIC Values of the Tested Strain

**FIGURE 1:**
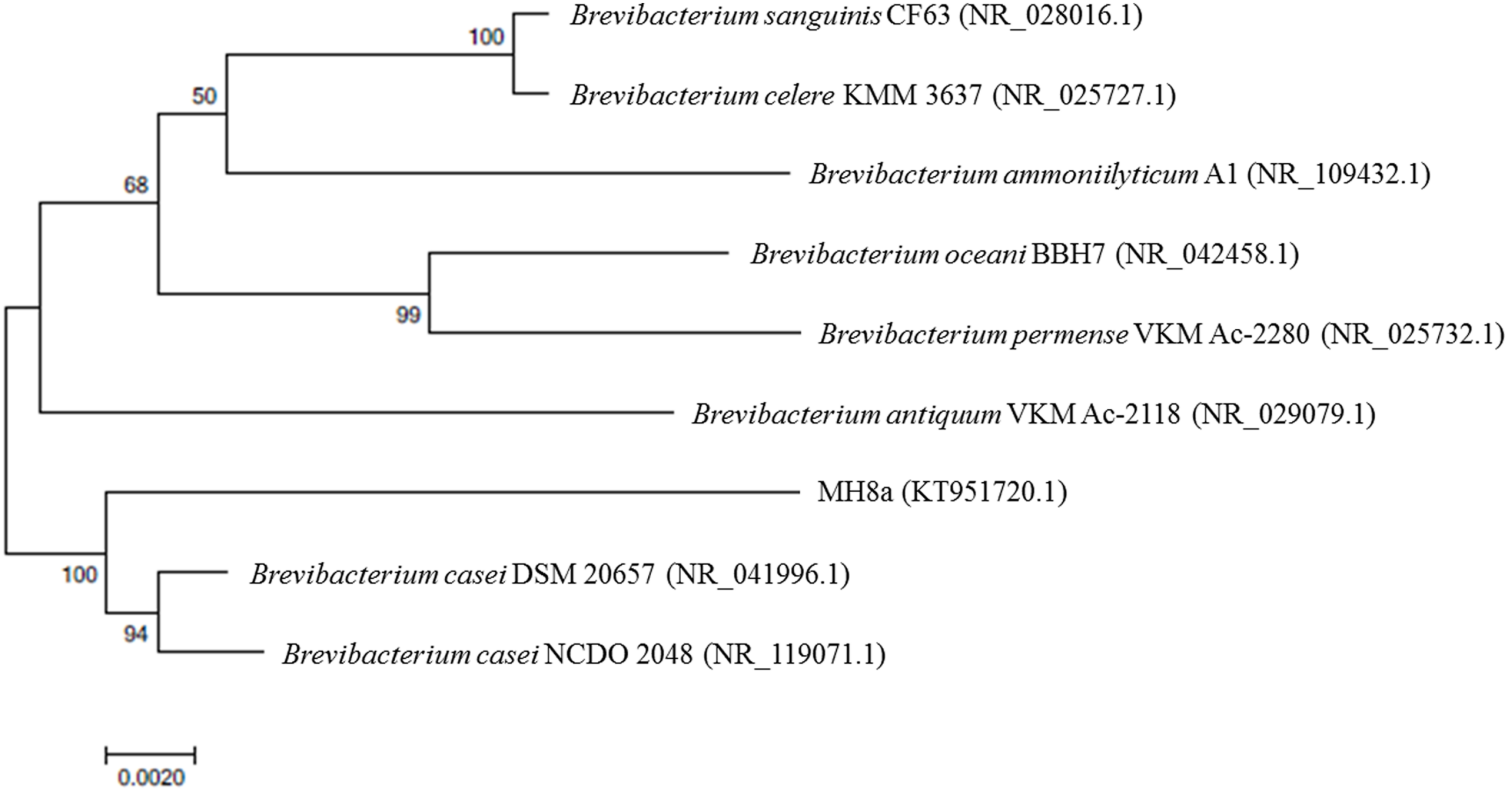
**Neighbor–joining phylogenetic tree of bacteria based on a comparison of 16S rRNA gene sequence.** Bootstrap values are indicated at the branches from 1,000 replications. GenBank accession numbers are given in brackets.

Based on the MIDI-FAME method and 16S rRNA gene sequence analysis, the isolated strain was identified as *B. casei* and designed as MH8a strain. The phylogenetic analysis showed that the 16S rDNA sequence of *B. casei* MH8a (KT951720.1) had a 98 and 97% identity with strains *B. casei* DSM 20657 (NR_041996.1) and *B. casei* NCDO 2048 (NR_119071.1), respectively (**Figure 1**).

The MIC values for Zn, Cd, and Cu reached values of 7, 1.5, and 6 mM, respectively.

### Biochemical Characteristic of MH8a (PGP Traits)

Among the tested mechanisms that were considered to be potentially responsible for the support of plant growth and heavy metal phytoextraction, MH8a was able to produce ammonia, hydrocyanic acid and indole 3-acetic acid at a concentration of 3.44 ± 0.09 μg IAA mL^–1^ of medium. The MH8a showed the activity of ACC deaminase at a level of 53.6 ± 1.47 nmol α-ketobutyrate mg^–1^ h^–1^. *B. casei* MH8a was not able to solubilize Ca_3_(PO_4_)_2_ in an NBRIP medium to produce siderophores on a CAS medium.

### Effect of Bacteria Inoculation on Plant Biomass and Metal Accumulation

**FIGURE 2:**
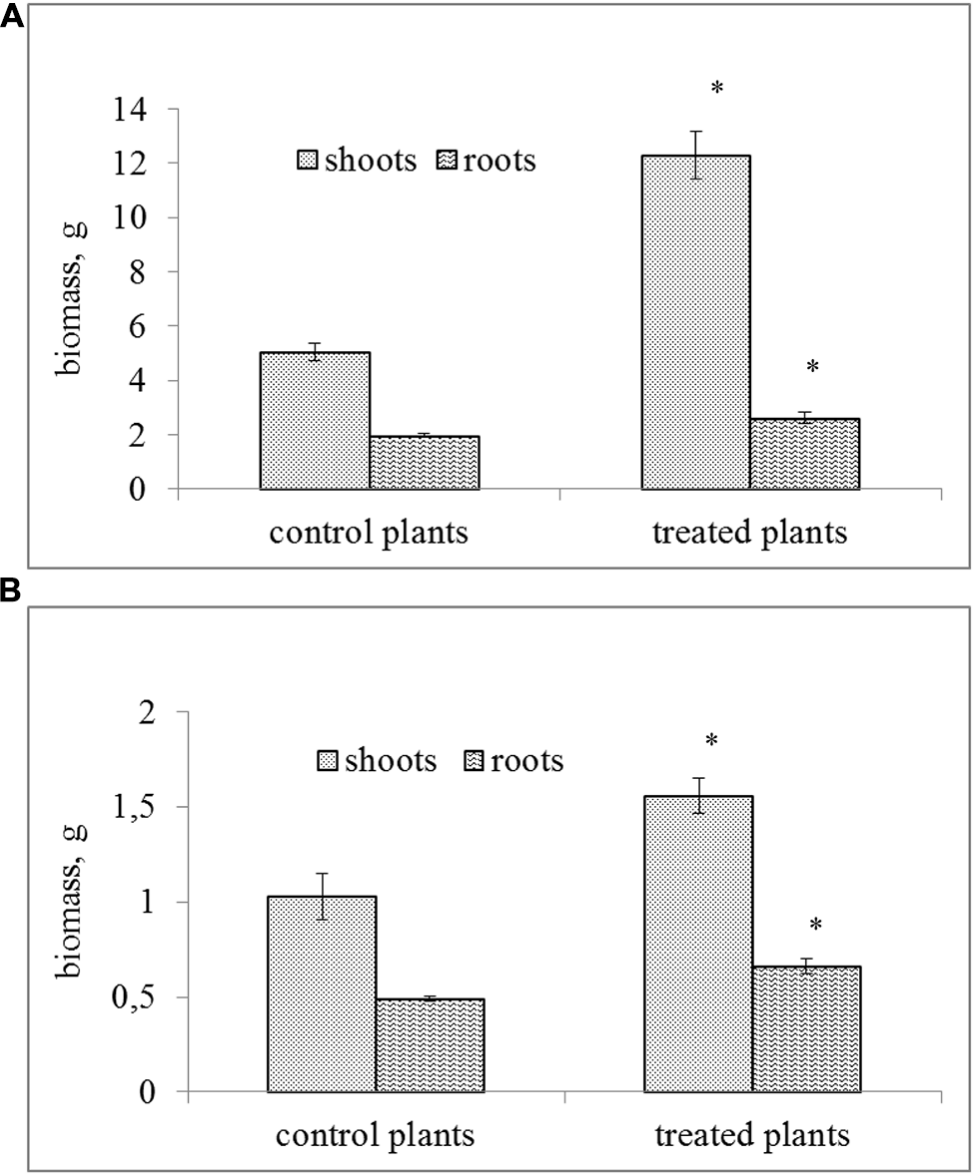
**The fresh (A) and dry (B) biomass of the shoots and roots of *Sinapis alba* L. growing in soil inoculated with the MH8a strain (mean ± SD, *n* = 3).** ^∗^Indicates singnificant differences at the 0.01 probability level.

The inoculation of soil with the tested bacterial strain resulted in a significant increase of the biomass of *S. alba* as compared to the control plants (**Figure 2**). MH8a enhanced the fresh and dry weight of shoots by 144 and 51%, respectively, and significantly increased (about 33%) the fresh and dry weight of roots as compared to the control plants.

The concentrations of Zn, Cd, and Cu were significantly higher in plants growing in soil inoculated with MH8a in comparison with the control plants (**Table 1**). MH8a led to a significant increase in Zn (87%), Cd (207%), and Cu (39%) accumulation in shoot tissues. The tested strain also caused a significant increase in the accumulation of metals in the roots of *S. alba*, where the Zn, Cd, and Cu accumulation was 85, 87, and 122% higher as compared to the control plants, respectively.

**Table 1:** Heavy metal accumulation in the shoots and roots of *Sinapis alba* L. growing in the control soil and soil inoculated with *Brevibacterium casei* MH8a.

Soil treatment		Metal accumulation (mg kg^–1^)		
		Zn	Cd	Cu
Control plants	Shoots	736 @ 21	6.2 @ 0.7	5.1 @ 0.6
	Roots	721.3 @ 36	11.1 @ 0.5	23.1 @ 0.6
Treated plants	Shoots	1375ˆ* @ 31	19.2ˆ* @ 0.4	7.1ˆ* @ 0.3
	Roots	1331ˆ* @ 22	20.9ˆ* @ 0.5	51.3ˆ* @ 1.2

^∗^*p < 0.01.*

As is shown by the values of TF, the inoculation of soil with MH8a resulted in a markedly higher Cd translocation from root to shoot. The value of TF for Cd in plants growing in soil treated with MH8a reached a value of 0.91, whereas in the control plants, it was significantly lower (0.55).

### Effect of the Bacterial Inoculant on the Structure of Bacterial Community

**FIGURE 3:**
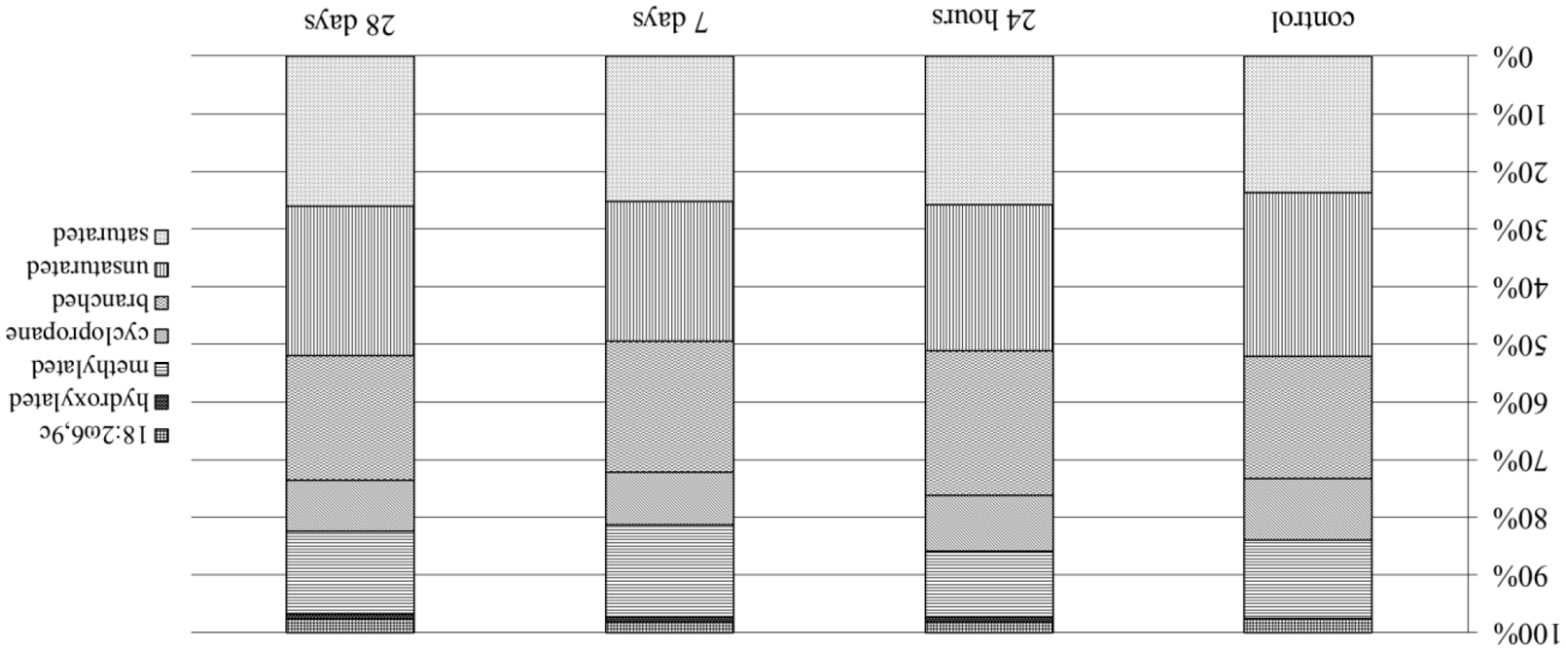
**Phospholipid fatty acid (PLFA) profiles derived from the non-inoculated control and soil inoculated with the MH8a strain**.

The inoculation of MH8a into the soil caused temporary changes in the PLFA profiles that were obtained from the treated soil as compared to the control soil (**Figure 3**). All PLFAs were divided into structural classes and among them hydroxylated, cycloprane, and unsaturated fatty acids were considered to be characteristic for Gram-negative bacteria. In turn, the branched fatty acids indicated the presence of Gram-positive bacteria. The inoculation of MH8a into soil caused an increase of branched fatty acids content (4.4%) in PLFA profiles after 24 h. Additionally, a decrease in the percentage of unsaturated fatty acids (3.3%) was also observed. At the same time, the amount of 18:2ω6,9c decreased from 2.5 to 1.9%. Seven days after soil treatment, the percentage of branched fatty acids decreased about 2.6% in the PLFA profiles as compared to samples at 24 h. At the same time, an increase in the percentage of methylated fatty acids was observed. Analysis of the PLFA profiles from 28 days samples (end of the experiment) showed a decrease in the percentage of branched fatty acids and an increase of 18:2ω6,9c as compared with the PLFA profiles from the 24 h and 7 days samples.

**FIGURE 4:**
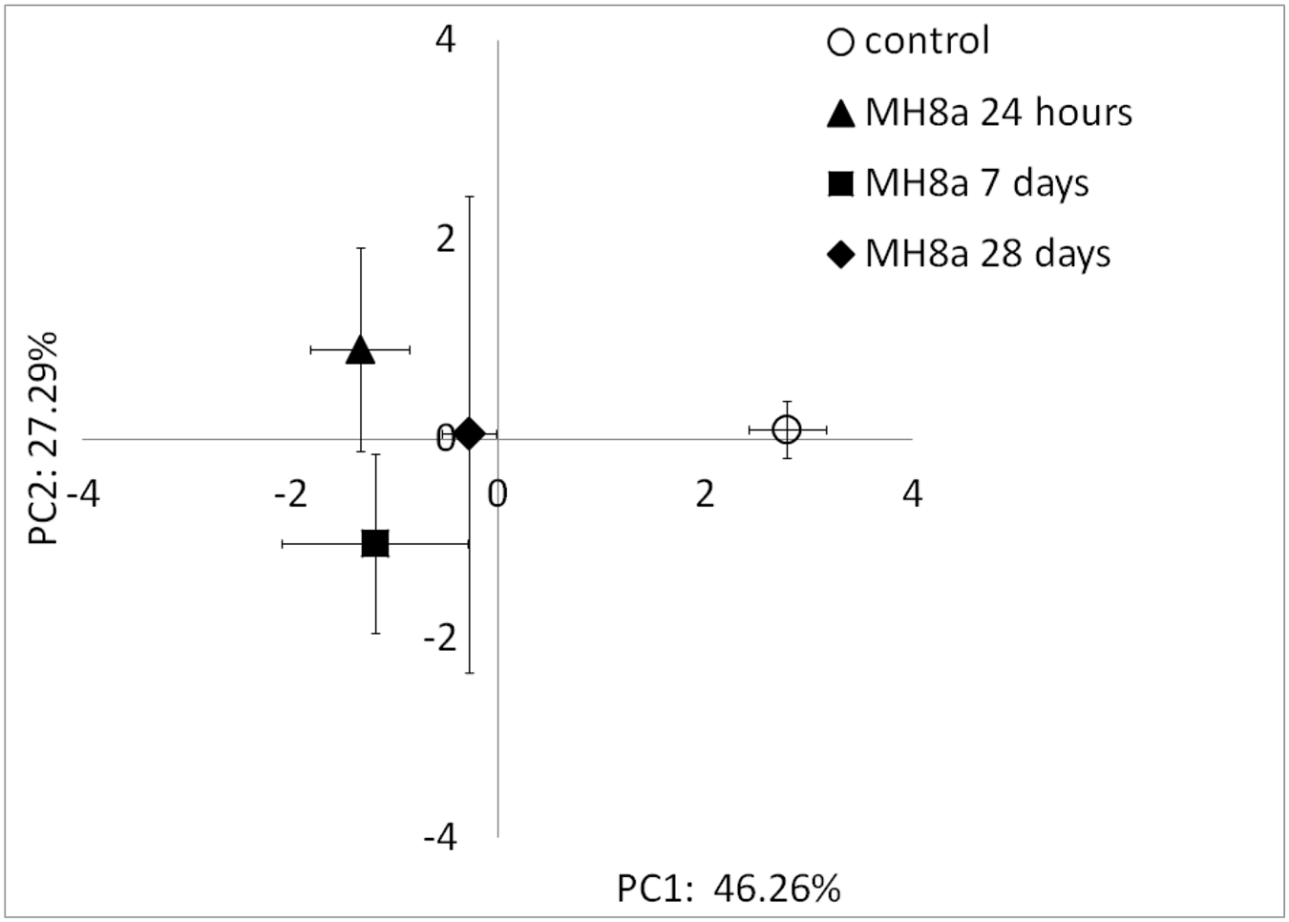
**Principal component analysis (PCA) plot of the PLFA profiles extracted from the non-inoculated control and soil inoculated with the MH8a strain.** Mean of the PCA-axes values of PLFA profiles ± SD (*n* = 3, *p* < 0.05).

Phospholipid fatty acid profiles differed significantly (*p* < 0.001) along PC1, which explained 46.26% of the total variability between the tested samples (**Figure 4**). All PLFA profiles extracted from the soil inoculated with the MH8a strain (24 h, 7 and 28 days) differed significantly as compared to the profiles obtained from the control soil. The most pronounced changes in the bacterial community structure were observed 24 h after soil inoculation with MH8a. Profiles at 24 h also differed significantly from those obtained for soil taken 28 days after soil inoculation with the tested strain. Among the tested samples, PLFA profiles from soil taken at the end of experiment (28 days) had the nearest location to the control samples, but the differences were still statistically significant.

### Survival of MH8a in Soil and its Ability to Colonize Plant Tissues

**FIGURE 5:**
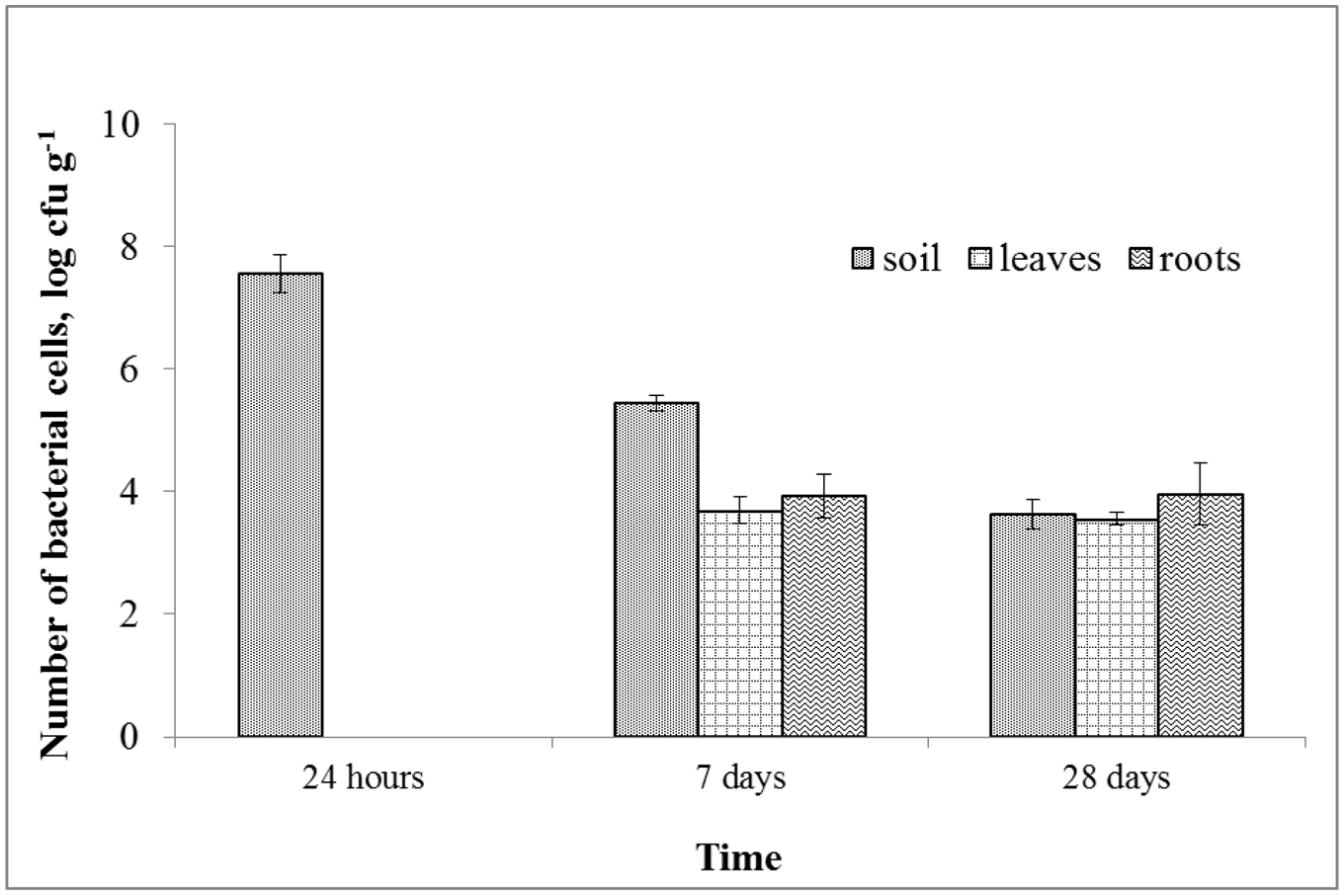
**The number of Brevibacterium casei MH8a cells isolated from soil and plant tissues 24 h, 7 and 28 days after soil inoculation with MH8a (mean ± SD, n = 3)**.

A rifampicin-resistant mutant of the MH8a strain was used for the assessment of its survival in soil and its ability to colonize *S. alba* tissues after soil inoculation. A gradual decrease in cell number in soil was observed during the experimental period (**Figure 5**). At the first sampling time, the number of MH8a cells decreased three-fold (to 3.6 × 10^7^ cfu g^–1^ dw of soil) as compared to the number of bacteria introduced into the soil (10^8^ cfu g^–1^ dw of soil). On the last sampling day (28 days), the number of MH8a cells in soil was 4 × 10^3^ cfu g^–1^ of dry soil. The plate inoculation with the plant’s suspension showed that no rifampicin-resistant bacteria were isolated from plant tissue 24 h after soil treatment with MH8a. Seven and 28 days after the introduction of MH8a into the soil, the number of bacteria that were isolated from the leaves of *S. alba* reached a value of 4.9 × 10^3^ and 3.6 × 10^3^ cfu g^–1^ of the dw of leaves, respectively. At the same sampling points, the number of MH8a cells in the roots of *S. alba* reached a value of 8.6 × 10^3^ and 9.2 × 10^3^ cfu g^–1^ of the dw of roots, respectively.

## Discussion

The plant–bacteria partnership can be applied to increase the phytoremediation efficiency of soil and water contaminated with organic and/or inorganic pollutants (Weyens et al., 2009; Khan et al., 2015). The results of many studies revealed that phytoextraction may be significantly enhanced by various bacteria including PGPB (Jiang et al., 2008; Rajkumar and Freitas, 2008; Dimkpa et al., 2009; Ma et al., 2009; Płociniczak et al., 2013a). Recently, attention has concentrated on the bioaugmentation of soil with bacteria that are characterized by metal resistance and plant growth-promoting traits (Ma et al., 2015).

In the present study, we describe the metal-tolerant bacterial strain *B. casei* MH8a, which was isolated from the rhizosphere of *S. alba* that was growing in heavy metal contaminated soil, as a potential agent in the enhancement of phytoextraction. Moreover, this strain was able to establish a close interaction with *S. alba* by colonizing its tissues.

Endophytic bacteria may improve plant tolerance to heavy metals and plant growth through several biological mechanisms. The beneficial effects of endophytic bacteria are generally attributed to the utilization of ACC, the production of IAA and siderophores and the solubilization of phosphates (Kolbas et al., 2015). Bacterial ACC deaminase can limit the level of the stress hormone ethylene in plants that are growing in harsh conditions thus increasing a plant’s growth. Additionally, the secretion of phytohormones such as IAA can lead to the formation of ACC, which in turn may be converted into ammonium and α-ketobutyrate by endophytic bacteria. The increase in the biomass and metal accumulation in plants that was observed in our studies may be connected with the biochemical features of the MH8a strain. Biochemical analysis of MH8a revealed the activity of several mechanisms that may potentially be responsible for the promotion of plant growth and indirectly for the enhancement of phytoextraction. The ACCD activity at the level of 53.6 ± 1.47 nmol α-ketobutyrate mg^–1^ h^–1^, IAA production (3.44 ± 0.09 μg IAA mL^–1^) as well as the release of ammonia and hydrocyanic acid allows the MH8a strain to be classified as a PGPB. It is very difficult to connect the positive effect of MH8a on plant growth and heavy metal phytoextraction with a specific bacterial mechanism. For example, it has been observed that a low level of ACCD activity, approximately ≥20 nmol α-ketobutyrate mg^–1^ protein h^–1^, is sufficient to enable a bacterium to grow on ACC and to act as a PGPB. Interestingly, organisms with a higher ACCD activity (300–400 nmol α-ketobutyrate mg^–1^ protein h^–1^) do not necessarily promote root elongation more than the strains that are characterized by a lower enzyme activity (Penrose and Glick, 2003). The secretion of siderophores and phosphate solubilization are also considered to be features of PGPB, but MH8a did not exhibit these activities. Such bacterial mechanisms are essential in niches that are poor in bioavailable forms of iron and phosphorus. During the phytoextraction experiment, symptoms of an Fe and P deficiency were not observed in *S. alba* indicating that bacteria and plants did not suffer from a lack of these nutrients.

The effect of MH8a on plant biomass was expressed in the significant increase of *S. alba* weight in the plants that were cultivated in soil treated with the tested strain. This result is in agreement with previous reports describing a greater biomass of *S. alba* growing in soil treated with *Enterobacter intermedius* MH8b and several *Pseudomonas* strains that had also been isolated from heavy metal contaminated soil (Płociniczak et al., 2013a,b). The positive impact of endophytic bacteria on plant biomass has been confirmed by several authors. For instance, Chen et al. (2014) found that the biomass of *Sedum alfredii* treated with the *Sphingomonas* SaMR12 strain was significantly higher as compared with a non-inoculated control. Similarly, Kolbas et al. (2015) confirmed the positive effect of endophytic bacteria (mainly from *Bacillus* and *Rhodococcus* genera) isolated from seeds of *Agrostis capilaris* on the biomass of inoculated plants grown in soil contaminated with Cu (13–114 mg kg^–1^). They found that at these Cu concentrations, endophytic inoculants increased shoot biomass between 1.6 and twofold compared to the control plants.

Plants that might be useful in the phytoextraction process, besides possessing a high biomass, should uptake and accumulate heavy metals with a high efficiency. The efficiency of the phytoextraction of metal-contaminated soil can be enhanced by metal-tolerant PGPE (Mastretta et al., 2009; Ma et al., 2011b). In our study we observed that the introduction of the multi-metal-resistant strain MH8a into soil resulted in a significantly higher accumulation of Zn, Cd, and Cu in the shoots and roots of *S. alba*. In comparison with the control plants, the amount of Zn, Cd, and Cu in the shoots of inoculated plants was 87, 207, and 39% higher, respectively. The positive role of metal-resistant PGPE in the enhancement of phytoextraction by *Sedum plumbizincicola* was reported earlier by Ma et al. (2015). Among the tested strains (*Bacillus pumilus* E2S2, *Bacillus* sp. E1S2, *Bacillus* sp. E4S1, *Achromobacter* sp. E4L5 and *Stenotrophomonas* sp. E1L), the most effective strain, *B. pumilus* E252, significantly increased the fresh and dry biomass of plants (37 and 32%, respectively), as well as the plant Cd uptake (43%). In other studies Ma et al. (2011b) showed that the PGPE *Pseudomonas* sp. A3R3 significantly increased the Ni accumulation in *Alyssum serpyllifolium* during microbial-assisted phytoremediation. Similarly, Mastretta et al. (2009) confirmed the positive effect of Cd-resistant *Sanguibacter* sp. on Cd phytoextraction by *Nicotiana tabacum*. Plants growing in soil treated with this strain accumulated a significantly higher amount of Cd, as compared to control plants. It is worth emphasizing that MH8a caused the higher translocation of Cd from the roots to the shoots of plants was confirmed by the higher value of the TF for cadmium (0.91) as compared to the control plants (0.56). This result in combination with the high TF for Zn (1.03) makes the system of white mustard and *B. casei* MH8a especially useful for the phytoextraction of soil contaminated with zinc and cadmium.

It is widely accepted that the inoculants that are used in bioaugmentation should not upset the microbial equilibrium in soil; however, very little is known about the impact of PGPB/PGPE on the indigenous microbial communities in soil. This is particularly important because most of the bacteria that are used in the bioaugmentation process are introduced directly into soil.

In our study the bacterial communities of both the control and inoculated soils were dominated by Gram-negative bacteria. As was indicated by PLFA analysis, the introduction of MH8a caused temporary changes in the structure of the bacterial populations in inoculated soils. The temporary changes in the percentage of branched fatty acids in the PLFA profiles probably resulted from the introduction of the Gram-positive MH8a strain. However, the changes decreased over the period of experiment. This was observed because MH8a was isolated from the same heavy-metal polluted soil that was used in the phytoextraction experiment and was a member of the autochthonous population of microorganisms.

The strain used in our study had the ability to enter a plant’s tissues and become an endophyte. One of the problems connected with the use of endophytes as an inoculum is their poor survival in soil and weak recolonization of plants, which are caused by harsh soil conditions and competition between bacterial species. It seems that the bioaugmentation of soil with metal-tolerant PGPB, which have originated from the rhizosphere or bulk soil and are able to colonize plant tissue is an alternative that may reduce the risk of the poor survival of an inoculant.

For the effective enhancement of phytoextraction by bacteria, the bacteria that are introduced should interact closely with plants and exhibit the ability to colonize the rhizosphere and/or interior of plant tissues. The plant apoplast offers different growth conditions, and therefore, different strains originating from rhizosphere can efficiently colonize the plant interior and become endophytes (Khan et al., 2015).

The strain that was used in this study was able to colonize both the rhizosphere soil and plant tissues and this interaction supported the phytoextraction of heavy metals. Seven days after the inoculation of the soil with the MH8a strain, rifampicin-resistant bacteria were detected in the rhizosphere as well as in the roots and leaves of *S. alba*. Similar experiments on bacterial survival in the rhizosphere soil and tissues of apple seedlings was evaluated by Ju et al. (2014), who studied the ability of *Bacillus subtilis* Y-1 to colonize and protect apple seedlings against *Fusarium oxysporum*. Using the plating method, the authors showed that the rifampicin-resistant strain of Y-1 could colonize the rhizosphere and plant tissues within 30 days. The colonization of potato plants by rifampicin-resistant strains of *Paenibacillus* sp. E119 and *Methylobacterium mesophilicum* SR1.6/6 was tested by Andreote et al. (2010). The authors emphasized the role of the shifts in the compositions of plant-associated communities after the successful colonization of plant tissues by the bacterial inoculant. These shifts may lead to differences in the plant’s metabolism thereby influencing plant growth and crop yields.

## Conclusion

A metal-tolerant *B. casei* MH8a strain, due to the activity of several mechanisms that are considered to be important for plant growth-promotion, has the potential to enhance the phytoextraction of heavy metals from contaminated soil. Additionally, MH8a showed a high survival rate after introduction into the soil and was able to colonize the internal tissues of *S. alba*. These features indicate that MH8a in combination with *S. alba* can be regarded as an effective tool for the phytoextraction of Cd and Zn.

## Author Contributions

All authors listed, have made substantial, direct and intellectual contribution to the work, and approved it for publication.

## Conflict of Interest Statement

The authors declare that the research was conducted in the absence of any commercial or financial relationships that could be construed as a potential conflict of interest. The reviewer RSO and handling Editor declared their shared affiliation, and the handling Editor states that the process nevertheless met the standards of a fair and objective review.

## References

[B1] Abou-Shanab R. A. Ghanem K. Ghanem N. Al-Kolaibe A. (2008). The role of bacteria on heavy-metal extraction and uptake by plants growing on multi-metal-contaminated soils. *World J. Microbiol. Biotechnol.* 24 253–262. 10.1007/s11274-007-9464-x

[B2] Ali H. Khan E. Sajad M. A. (2013). Phytoremediation of heavy metals – Concepts and applications. *Chemosphere* 91 869–881. 10.1016/j.chemosphere.2013.01.075 23466085

[B3] Andreote F. D. da Rocha U. N. Araújo W. L. Azevedo J. L. van Overbeek L. S. (2010). Effect of bacterial inoculation, plant genotype and developmental stage on root-associated and endophytic bacterial communities in potato (*Solanum tuberosum*). *Anton. Leeuw. Int. J. G.* 97 389–399. 10.1007/s10482-010-9421-9 PMC2847171 20352404

[B4] Arshad M. Saleem M. Hussain S. (2007). Perspectives of bacterial ACC deaminase in phytoremediation. *Trends Biotechnol.* 25 356–362. 10.1016/j.tibtech.2007.05.005 17573137

[B5] Becerra-Castro C. Kidd P. S. Prieto-Fernández Á Weyens N. Acea M. J. Vangronsveld J. (2011). Endophytic and rhizoplane bacteria associated with *Cytisus striatus* growing on hexachlorocyclohexane-contaminated soil: isolation and characterization. *Plant Soil* 340 413–433. 10.1007/s11104-010-0613-x

[B6] Bradford M. (1976). A rapid and sensitive method for the quantitation of microgram quantities of protein utilizing the principle of protein-dye binding. *Anal. Biochem.* 72 248–254. 10.1016/0003-2697(76)90527-3 942051

[B7] Bric J. M. Bostock R. M. Silverstone S. E. (1991). Rapid in situ assay for indoleacetic acid production by bacteria immobilized on a nitrocellulose membrane. *Appl. Environ. Microbiol.* 57 535–538. 16348419 10.1128/aem.57.2.535-538.1991 PMC182744

[B8] Cappuccino J. C. Sherman N. (1992). *Microbiology: A Laboratory Manual*, 3rd Edn. New York, NY: Benjamin Cummings Publisher, 125–179.

[B9] Chen B. Shen J. Zhang X. Pan F. Yang X. Feng Y. (2014). The endophytic bacterium, *Sphingomonas* SaMR12, improves the potential for zinc phytoremediation by its host *Sedum alfredii*. *PLoS ONE* 9:e106826 10.1371/journal.pone.0106826 PMC4157784 25198772

[B10] Chen L. Luo S. Xiao X. Guo H. Chen J. Wan Y. (2010). Application of plant growth-promoting endophytes (PGPE) isolated from *Solanum nigrum* L. for phytoextraction of Cd-polluted soils. *Appl. Soil Ecol.* 46 383–389. 10.1016/j.apsoil.2010.10.003

[B11] De-Bashan L. E. Hernandez J. P. Bashan Y. (2012). The potential contribution of plant growth-promoting bacteria to reduce environmental degradation–a comprehensive evaluation. *Appl. Soil Ecol.* 6 171–189. 10.1016/j.apsoil.2011.09.003

[B12] Dimkpa C. O. Merten D. Svatos A. Büchel G. Kothe E. (2009). Siderophores mediate reduced and increased uptake of cadmium by *Streptomyces tendae* F4 and sunflower (*Helianthus annuus* ), respectively. *J. Appl. Microbiol.* 107 1687–1696. 10.1111/j.1365-2672.2009.04355.x 19457036

[B13] Glick B. R. (2005). Modulation of plant ethylene levels by the bacterial enzyme ACC deaminase. *FEMS Microbiol. Lett.* 251 1–7. 10.1016/j.femsle.2005.07.030 16099604

[B14] Glick B. R. (2010). Using soil bacteria to facilitate phytoremediation. *Biotechnol. Adv.* 28 367–374. 10.1016/j.biotechadv.2010.02.001 20149857

[B15] Gravel V. Antoun H. Tweddell R. J. (2007). Growth stimulation and fruit yield improvement of greenhouse tomato plants by inoculation with *Pseudomonas putida* or *Trichoderma atroviride*: possible role of indole acetic acid (IAA). *Soil Biol. Biochem.* 39 1968–1977. 10.1016/j.soilbio.2007.02.015

[B16] Honma M. Shimomura T. (1978). Metabolism of 1-aminocylcopropane carboxylic acid. *Agric. Biol. Chem.* 42 1825–1831. 10.1080/00021369.1978.10863261

[B17] Jiang C. Y. Sheng X. F. Qian M. Wang Q. Y. (2008). Isolation and characterization of a heavy metal-resistant *Burkholderia* sp. from heavy metal-contaminated paddy field soil and its potential in promoting plant growth and heavy metal accumulation in metal-polluted soil. *Chemosphere* 72 157–164. 10.1016/j.chemosphere.2008.02.006 18348897

[B18] Jing X. B. He N. Zhang Y. Cao Y. R. Xu H. (2012). Isolation and characterization of heavy-metal-mobilizing bacteria from contaminated soils and their potential in promoting Pb, Cu and Cd accumulation by *Coprinus comatus*. *Can. J. Microbiol.* 58 45–53. 10.1139/W11-110 22181009

[B19] Jing Y. Zhen-li H. Yang X. (2007). Role of soil rhizobacteria in phytoremediation of heavy metal contaminated soils. *J. Zhejiang Univ. Sci. B* 8 192–207. 10.1631/jzus.2007.B0192 17323432 PMC1810380

[B20] Ju R. Zhao Y. Li J. Jiang H. Liu P. Yang T. (2014). Identification and evaluation of a potential biocontrol agent *Bacillus subtilis*, against *Fusarium* sp. in apple seedlings. *Ann. Microbiol.* 64 377–383. 10.1007/s13213-013-0672-3

[B21] Juwarkar A. A. Dubey K. V. Nair A. Singh S. K. (2008). Bioremediation of multi-metal contaminated soil using biosurfactant –a novel approach. *Indian J. Microbiol.* 48 142–146. 10.1007/s12088-008-0014-5 23100708 PMC3450211

[B22] Khan M. U. Sessitsch A. Harris M. Fatima K. Imran A. Arslan M. (2015). Cr-resistant and endophytic bacteria associated with *Prosopis juliflora* and their potential as phytoremediation enhancing agents in metal-degraded soils. *Front. Plant Sci.* 5:755 10.3389/fpls.2014.00755 PMC4284999 25610444

[B23] Kolbas A. Kidd P. Guinberteau J. Jaunatre R. Herzig R. Mench M. (2015). Endophytic bacteria take the challenge to improve Cu phytoextraction by sunflower. *Environ. Sci. Pollut. Res.* 22 5370–5382. 10.007/s11356-014-4006-1 25561255

[B24] Kukla M. Płociniczak T. Piotrowska-Seget Z. (2014). Diversity of endophytic bacteria in *Lolium perenne* and their potential to degrade petroleum hydrocarbons and promote plant growth. *Chemosphere* 117 40–46. 10.1016/j.chemosphere.2014.05.055 24954306

[B25] Li H. Wei D. Shen M. Zhou Z. (2012). Endophytes and their role in phytoremediation. *Fungal Divers.* 54 11–18. 10.1007/s13225-012-0165-x

[B26] Li K. Ramakrishna W. (2011). Effect of multiple metal resistant bacteria from contaminated lake sediments on metal accumulation and plant growth. *J. Hazard. Mater.* 189 531–539. 10.1016/j.jhazmat.2011.02.075 21420236

[B27] Lorck H. (1948). Production of hydrocyanic acid by bacteria. *Physiol. Plant.* 1 142–146. 10.1111/j.1399-3054.1948.tb07118.x

[B28] Ma Y. Oliveira R. S. Nai F. Rajkumar M. Luo Y. Rocha I. (2015). The hyperaccumulator *Sedum plumbizincicola* harbors metal-resistant endophytic bacteria that improve its phytoextraction capacity in multi-metal contaminated soil. *J. Environ. Manage.* 156 62–69. 10.1016/j.jenvman.2015.03.024 25796039

[B29] Ma Y. Prasad M. N. V. Rajkumar M. Freitas H. (2011a). Plant growth promoting rhizobacteria and endophytes accelerate phytoremediation of metalliferous soils. *Biotechnol. Adv.* 29 248–258. 10.1016/j.biotechadv.2010.12.001 21147211

[B30] Ma Y. Rajkumar M. Luo Y. Freitas H. (2011b). Inoculation of endophytic bacteria on host and non-host plants – effects on plant growth and Ni uptake. *J. Hazard. Mater.* 195 230–237. 10.1016/j.jhazmat.2011.08.034 21872991

[B31] Ma Y. Rajkumar M. Freitas H. (2009). Improvement of plant growth and nickel uptake by nickel resistant-plant-growth promoting bacteria. *J. Hazard. Mater.* 166 1154–1161. 10.1016/j.jhazmat.2008.12.018 19147283

[B32] Mastretta C. Taghavi S. van der Leile D. Mengoni A. Galardi F. Gonnelli C. (2009). Endophytic bacteria from seeds of *Nicotiana tabacum* can reduce cadmium phytotoxicity. *Int. J. Phytoremediat.* 11 251–267. 10.1080/15226510802432678

[B33] Pacwa-Płociniczak M. Płaza G. A. Poliwoda A. Piotrowska-Seget Z. (2014). Characterization of hydrocarbon-degrading and biosurfactant-producing *Pseudomonas* sp. P-1 strain as a potential tool for bioremediation of petroleum-contaminated soil. *Environ. Sci. Pollut. Res.* 21 9385–9395. 10.1007/s11356-014-2872-1 PMC4125813 24743958

[B34] Pawlik M. Piotrowska-Seget Z. (2015). Endophytic bacteria associated with *Hieracium piloselloides*: their potential for hydrocarbon-utilizing and plant growth-promotion. *J. Toxicol. Environ. Health A.* 78 860–870. 10.1080/15287394.2015.1051200 26167752

[B35] Pennanen T. Liski J. Bååth E. Kitunen V. Uotila J. Westman C. J. (1999). Structure of the microbial communities in coniferous forest soils in relation to site fertility and stand development stage. *Microbiol. Ecol.* 38 168–179. 10.1007/s002489900161 10441709

[B36] Penrose D. M. Glick B. R. (2003). Methods for isolating and characterizing ACC deaminase-containing plant growth-promoting rhizobacteria. *Physiol. Plant.* 118 10–15. 10.1034/j.1399-3054.2003.00086.x 12702008

[B37] Płociniczak T. Kukla M. Wkatroba R. Piotrowska-Seget Z. (2013a). The effect of soil bioaugmentation with strains of *Pseudomonas* on Cd, Zn and Cu uptake by *Sinapis alba* L. *Chemosphere* 91 1332–1337. 10.1016/j.chemosphere.2013.03.008 23561856

[B38] Płociniczak T. Sinkkonen A. Romantschuk M. Piotrowska-Seget Z. (2013b). Characterization of *Enterobacter intermedius* MH8b and its use for the enhancement of heavy metals uptake by *Sinapis alba* L. *Appl. Soil Ecol.* 63 1–7. 10.1016/j.apsoil.2012.09.009

[B39] Rajkumar M. Ae N. Freitas H. (2009a). Endophytic bacteria and their potential to enhance heavy metal phytoextraction. *Chemosphere* 77 153–160. 10.1016/j.chemosphere.2009.06.047 19647283

[B40] Rajkumar M. Prasad M. N. V. Freitas H. Ae N. (2009b). Biotechnological applications of serpentine soil bacteria for phytoremediation of heavy metals. *Crit. Rev. Biotechnol.* 29 120–130. 10.1080/07388550902913772 19514893

[B41] Rajkumar M. Freitas H. (2008). Influence of metal resistant-plant growth-promoting bacteria on the growth of *Ricinus communis* in soil contaminated with heavy metals. *Chemosphere* 71 834–842. 10.1016/j.chemosphere.2007.11.038 18164365

[B42] Rathnayake I. V. N. Megharaj M. Krishnamurti G. S. R. Bolan N. S. Naidu R. (2013). Heavy metal toxicity to bacteria – Are the existing growth media accurate enough to determine heavy metal toxicity? *Chemosphere* 90 1195–1200. 10.1016/j.chemosphere.2012.09.036 23040649

[B43] Saleh S. S. Glick B. R. (2001). Involvement of gacS and rpoS in enhancement of the plant growth-promoting capabilities of *Enterobacter cloacae* CAL2 and UW4. *Can. J. Microbiol.* 47 698–705. 10.1139/w01-072 11575495

[B44] Salt D. E. Smith R. D. Raskin I. (1998). Phytoremediation. *Annu. Rev. Plant Physiol. Mol. Biol.* 49 643–668. 10.1146/annurev.arplant.49.1.643 15012249

[B45] Schwyn B. Neilands J. B. (1987). Universal chemical assay for the detection and determination of siderophores. *Anal. Biochem.* 160 47–56. 10.1016/0003-2697(87)90612-9 2952030

[B46] Sessitsch A. Kuffner M. Kidd P. Vangronsveld J. Wenzel W. W. Fallmann K. (2013). The role of plant-associated bacteria in the mobilization and phytoextraction of trace elements in contaminated soils. *Soil Biol. Biochem.* 60 182–194. 10.1016/j.soilbio.2013.01.012 23645938 PMC3618436

[B47] Shoebitz M. Ribaudo C. M. Pardo M. A. Cantore M. L. Ciampi L. Curá J. A. (2009). Plant growth promoting properties of a strain of *Enterobacter ludwigii* isolated from *Lolium perenne* rhizosphere. *Soil Biol. Biochem.* 41 1768–1774. 10.1016/j.soilbio.2007.12.031

[B48] Sun K. Liu J. Jin L. Gao Y. (2014). Utilizing pyrene-degrading endophytic bacteria to reduce the risk of plant pyrene contamination. *Plant Soil* 374 251–262. 10.1007/s11104-013-1875-x

[B49] Thavasi R. Marchant R. Banat I. M. (2014). “Biosurfactant applications in agriculture,” in *Biosurfactants: Production and Utilization – Processes, Technologies and Economics*, eds Kosaric N Sukan F. V. (Boca Raton, FL: CRC Press), 313–325.

[B50] Weyens N. van der Leile D. Taghavi S. Newman L. Vangronsveld J. (2009). Exploiting plant-microbe partnership to improve biomass production and remediation. *Trends Biotechnol.* 27 591–598. 10.1016/j.tibtech.2009.07.006 19683353

[B51] Yoon J. Cao X. Zhou Q. Ma L. Q. (2006). Accumulation of Pb, Cu, and Zn in native plants growing on a contaminated Florida site. *Sci. Total Environ.* 368 456–464. 10.1016/j.scitotenv.2006.01.016 16600337

